# Cost-effectiveness of Sick Leave Policies for Health Care Workers with Influenza-like Illness, Brazil, 2009

**DOI:** 10.3201/eid1708.101546

**Published:** 2011-08

**Authors:** Nancy Val y Val P. Mota, Renata D. Lobo, Cristiana M. Toscano, Antonio C. Pedroso de Lima, M. Beatriz Souza Dias, Helio Komagata, Anna S. Levin

**Affiliations:** Author affiliations: Hospital das Clínicas, São Paulo, Brazil (N.V.V.P. Mota, R.D. Lobo, M.B. Souza Dias, H. Komagata, A.S. Levin);; Federal University of Goiás, Goiânia, Brazil (C.M. Toscano);; University of São Paulo, São Paulo (A.C. Pedroso de Lima, A.S. Levin)

**Keywords:** Influenza A virus, H1N1 subtype, pandemic (H1N1) 2009, sick leave, costs, cost analysis, health care workers, viruses, Brazil, influenza, research

## Abstract

We describe the effect of influenza-like illness (ILI) during the outbreak of pandemic (H1N1) 2009 on health care worker (HCW) absenteeism and compare the effectiveness and cost of 2 sick leave policies for HCWs with suspected influenza. We assessed initial 2-day sick leaves plus reassessment until the HCW was asymptomatic (2-day + reassessment policy), and initial 7-day sick leaves (7-day policy). Sick leaves peaked in August 2009: 3% of the workforce received leave for ILI. Costs during May–October reached R$798,051.87 (≈US $443,362). The 7-day policy led to a higher monthly rate of sick leave days per 100 HCWs than did the 2-day + reassessment policy (8.72 vs. 3.47 days/100 HCWs; p<0.0001) and resulted in higher costs (US $609 vs. US $1,128 per HCW on leave). ILI affected HCW absenteeism. The 7-day policy was more costly and not more effective in preventing transmission to patients than the 2-day + reassessment policy.

## Abstract 2

TOC Summary: Seven-day leave was more costly and no more effective than 2 days plus reevaluation.

During mid-April 2009, Mexico reported 1,918 cases of influenza-like illness (ILI) and 84 deaths. In July 2009, the World Health Organization (WHO) declared an influenza pandemic on the basis of widespread pandemic influenza A (H1N1) 2009 observed globally (*1*). On July 16, the Brazilian Ministry of Health notified transmission within the country and declared the epidemic to be widespread (*2*). During the 2009–10 season, pandemic (H1N1) 2009 was the main contributor to influenza infections.

In 2009, WHO reported 12,799 deaths from pandemic (H1N1) 2009. South America was affected during the winter season (June–September). Brazil reported 48,978 confirmed cases of pandemic (H1N1) 2009, with 2,051 deaths (*3*). In São Paulo, the most populated state in the country (≈40 million inhabitants), the reported incidence was higher than anywhere else in the country (15.17 cases/100,000 inhabitants); 479 persons died (*4*).

Hospital das Clínicas (HC), the largest hospital in Brazil, was assigned by the State Health Department as 1 of the reference hospitals for persons with severe pandemic (H1N1) 2009 in the city of São Paulo. During the pandemic, specific sick leave policies were instituted at HC for health care workers (HCWs) who had influenza.

Considerable concern exists among HCWs about the risks of working during an influenza epidemic. Although they feel responsible to care for patients, they also are concerned about their own and their families’ health (*5**,**6*). Transmission of influenza from HCWs to patients under their care is also a concern (*7*). Isolation precautions needed to prevent transmission of pandemic (H1N1) 2009 virus were heavily debated, and recommendations from the US Centers for Disease Control and Prevention (Atlanta, GA, USA) and WHO conflicted (*8*). Guidance on appropriate sick leave policies to avoid transmission from HCWs varies and is not well established (*9*).

Interim guidelines for protecting HCWs from pandemic (H1N1) 2009 (*9*) suggest that HCWs in whom fever and respiratory symptoms develop should be excluded from work for at least 24 hours after defervescence. HCWs caring for severely immunocompromised patients should be reassigned or excluded from work for 7 days after symptom onset or until resolution of symptoms, whichever is longer.

Workforces at large tertiary care hospitals functioning as reference hospitals for persons with influenza may be substantially affected during pandemics, particularly in regard to absenteeism and associated costs. Effectiveness and costs of sick leave policies should be evaluated to guide hospital managers and public health officials, particularly during epidemics and pandemics.

The objectives of this study were to describe the effects of ILI during the pandemic (H1N1) 2009 outbreak on HCW absenteeism and the associated costs. Furthermore, we aimed to compare effectiveness and cost of 2 policies for HCW sick leave during the first wave of pandemic (H1N1) 2009 in a large urban tertiary care hospital.

## Methods

### Location and Setting

The study was conducted at HC, in the city of São Paulo, São Paulo state, Brazil. São Paulo is among the largest cities in Latin America, with 11 million inhabitants. HC, a tertiary care teaching hospital complex affiliated with the University of São Paulo, is a government hospital predominantly for patients covered by the publicly funded Brazilian National Health Service. During the outbreak of pandemic (H1N1) 2009 in Brazil, HC was assigned by the State of São Paulo Health Department as a state reference hospital for persons with severe pandemic (H1N1) 2009.

HC has ≈2,000 beds distributed in 7 institutes. The Central Institute (main building) has 894 beds, including 100 intensive care unit (ICU) beds. The other centers are the Heart Institute (444 beds); Orthopedics Institute (152 beds); Children’s Institute (174 beds); Psychiatry and Neurosurgery Institute (90 beds); Cancer Institute (213 beds); and Radiology Institute, without inpatients, which serves all other institutes. Although also part of the HC complex, the administration building, 2 long-term care facilities (234 beds total), and 1 outpatient rehabilitation center were not included in this study.

### Influenza Triage, Diagnosis, and Treatment

As recommended by international guidelines, triage for suspected influenza cases was put in place at all of HC´s institutes. Triage occurred in the existing emergency departments in the Central, Heart, and Children’s Institutes. Persons with confirmed influenza were then referred to designated units within HC for hospitalization. Included in the Central Institute were an ICU (7 beds), an influenza-specific ward (11 beds for semi-intensive care), and a specific ward for pregnant influenza patients (15 beds). In the Children’s Institute, an ICU (2 beds) , a ward (14 beds), and 2 rooms at the emergency department were assigned. Later into the influenza season (August 2009), 2 additional influenza-specific wards were designated in the Heart Institute, with 16 rooms and 32 beds. Although persons with influenza should have been hospitalized preferentially in the above-designated units, a few patients in whom influenza was only suspected after admission and who could not be transferred were hospitalized in nondesignated units.

Laboratory testing for confirmatory diagnosis was conducted for all hospitalized persons suspected to have influenza. Rapid diagnostic tests for influenza were not performed during the pandemic. Before July 2, 2009, samples were collected and sent to the Adolfo Lutz Reference Laboratory, where real-time PCR (rt-PCR) testing was conducted. By July 2, 2009, HC Central Hospital Laboratory started the influenza A (H1N1) rt-PCR. Although available for patients admitted to the hospital, laboratory confirmation of pandemic (H1N1) 2009 infection was not available for HCWs. Thus, suspected cases among HCWs were triaged according to the attending physician’s evaluation. The rt-PCR for suspected influenza in HCWs was made available only on August 24, 2009. The PCR protocol in place was developed by the US Centers for Disease Control and Prevention (*10*).

During the entire pandemic period, active surveillance for nosocomial pandemic (H1N1) 2009 was performed by the infection control teams. A suspected influenza case was defined, according to national guidelines (*11*), as fever and respiratory symptoms such as cough or sore throat in the absence of other diagnoses, and confirmed cases were defined as those with positive test results for pandemic (H1N1) 2009.

### Infection Control Policies

Following global and national guidelines (*8**,**12*), infection control policies for prevention and control of nosocomial transmission of influenza were in place in the hospital. These included contact and droplet precautions taken by HCWs during the care of patients with suspected and confirmed influenza and use of N95 masks only during aerosol-generating procedures.

HC has a centralized occupational health service for workers of all institutes. We assessed ILI in and ILI-associated sick leave for HCWs who are overseen by the hospital’s occupational health service, i.e., physicians; nurses and nurse assistants; and pharmacy, nutrition, laboratory, and administration workers. Cleaning and security services are furnished by third-party service providers; therefore, their staff are not overseen by HC’s occupational health service.

Starting on July 16, 2009, in HC institutes except the Heart Institute, an HCW with suspected influenza received an initial 2-day leave and was reassessed every 2 days until asymptomatic, when he or she returned to work (2-day leave + reassessment policy). In the Heart Institute, a different sick leave policy was adopted in which an HCW with suspected pandemic (H1N1) 2009 infection received a 7-day leave (7-day leave policy). During the prepandemic period, sick leave for respiratory infection was determined on a case-by-case basis after evaluation by a physician (Figure).

**Figure:**
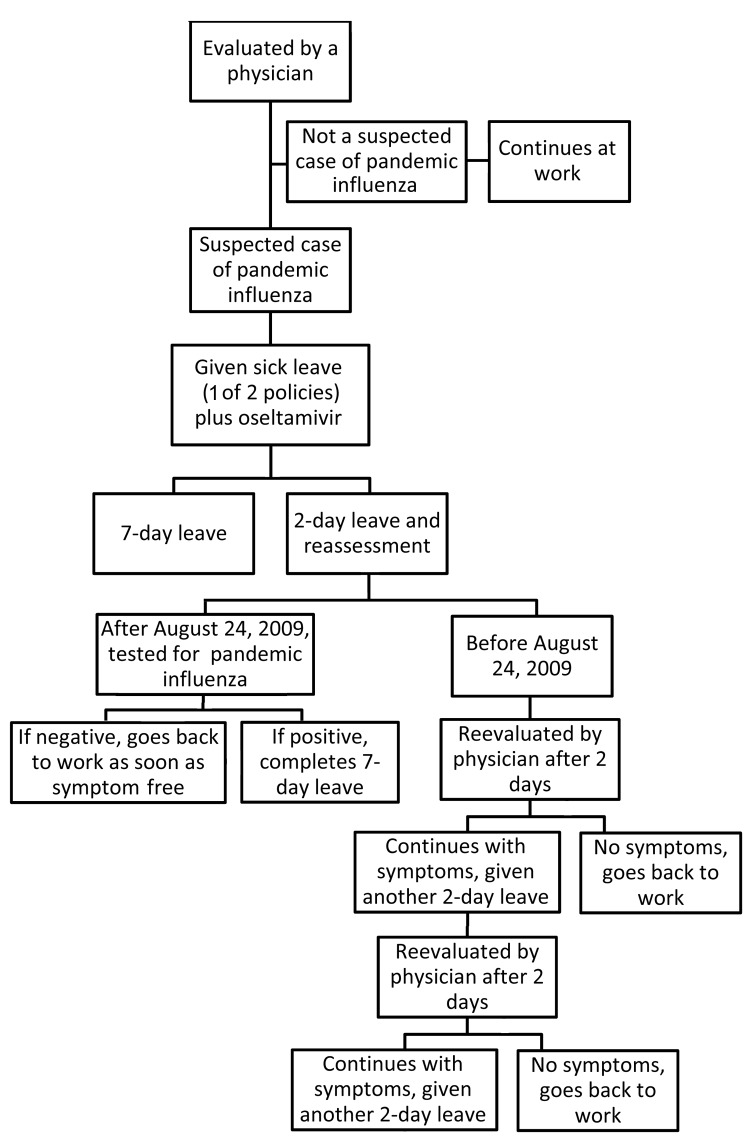
Schematic representation of the sequence of events that occurred each time symptoms consistent with influenza developed in a health care worker during the pandemic (H1N1) 2009 outbreak, Hospital das Clínicas, São Paulo, Brazil, May–October, 2009.

### Study Design

We retrospectively evaluated the effect of ILI on HCW absenteeism during the pandemic (H1N1) 2009 outbreak. The main outcome of interest was HCW absenteeism.

We estimated the economic effects of HCW-associated illness from the hospital´s perspective. Costs were estimated retrospectively. Among the various techniques for measuring costs, 1 of the most commonly used is the accounting approach (the conventional costing method). This approach can be divided into 2 categories: the first uses detailed, bottom-up, step-down analyses of accounting to distribute shared costs across the activities considered (also called ingredients approach). The second uses a top-down approach, which makes less detailed estimates of high-level average costs on the basis of aggregate expenditure records. In this study, we used the bottom-up approach as we considered individual ingredients to estimate costs. We compared the effectiveness and cost of the 2 sick leave policies: initial 2-day + reassessment policy and 7-day.

### Study Period

The study was conducted during the first wave of pandemic (H1N1) 2009 in the Southern Hemisphere winter season (May–October 2009). The prepandemic period was defined as August 2008–April 2009. HCW was defined as a professional on the hospital’s payroll, even if the worker did not work directly with patients. This definition excluded students and residents/fellows, as well as workers of third-party service providers (cleaning and security).

### Data Analysis

We calculated the monthly number, average duration, and rate of influenza sick leaves issued in HC by institute and HCW profession. We determined the proportion of sick leaves taken for ILI in relation to the total number of sick leaves during the pandemic (H1N1) 2009 outbreak (May–October). Rate of influenza-associated sick leaves issued during the pandemic (H1N1) 2009 outbreak (August–October 2009) were compared with the prepandemic period (August–October 2008).

To assess the effectiveness of the 2 sick leave policies, we evaluated the number of nosocomial pandemic (H1N1) 2009 cases. We calculated the total number of sick leaves issued, their average duration, and total days of ILI-associated sick leave for HCWs working under the 7-day and 2-day + reassessment policies during the pandemic period. In addition, monthly rates of ILI-associated sick leave days per 100 HCWs were compared for the 2 sick leave policies by using χ^2^.

### Cost Analysis

The cost analysis considered only ILI and absenteeism in physicians, nurses, and nurse assistants because of the availability of information. Direct medical costs of diagnosis, treatment, and follow-up for HCWs with suspected and confirmed pandemic (H1N1) 2009 were estimated. Direct medical costs also included the cost of replacement HCWs during sick leave. Direct nonmedical costs assessed were transportation of symptomatic HCWs to and from HC for assessment. Indirect costs comprised productivity losses of HCWs during sick leave. Costs for outpatient treatment of HCWs suspected to have influenza comprised medical assessment by the occupational health clinic, specimen collection and rt-PCR (only after August 24, 2009) for laboratory diagnosis, and treatment.

We assumed that 100% of HCWs with suspected influenza received antiviral medication (oseltamivir) according to the official hospital guideline. Twenty percent received antimicrobial drugs (amoxicillin) for secondary bacterial infection, and 70% received symptomatic medication.

HCW hospitalization costs associated with pandemic (H1N1) 2009 were estimated by considering the average daily cost of hospitalization in HC. No information about complications or secondary bacterial infection in hospitalized HCWs with influenza was available for the cost analysis.

When evaluating cost of staff replacement, we assumed that 70% of nurses and nurse assistants and 50% of physicians on sick leave were replaced. We also assumed that HCWs under the 2-day + reassessment policy would return on average 1.5 times for reassessment, thus incurring medical consultations and transportation costs for 1.5 return visits to HC. Under the 7-day policy, we assumed that 10% of HCWs would return for medical consultations during leave; thus, only 10% of HCWs required transportation for reassessment.

We estimated productivity losses by considering the daily 12-hour average salaries for each HCW category based on an average of 5 years of work in HC. Wages and mandatory Social Security and health care contributions were considered. Monthly salary was Brazilian real (R$) 3,071.54 for nurses working 30 h/week; R$2,541.55 for physicians working 20 h/week; and R$1,571.80 for nurse assistants working 30 h/week. One US dollar was equivalent to ≈R$1.80.

We also recalculated the costs for each sick leave policy on the basis of a lowest cost scenario and a highest cost scenario. For the lowest cost scenario, we assumed that HCWs on leave would have returned on average 0.5 times for reevaluation if under the 2-day + reassessment policy and would not have returned for the 7-day policy. We also assumed that 5% of HCWs received antimicrobial drugs and 20% received treatment for symptoms. Staff replacement was assumed to have been 30% for nurses and nurse assistants and 10% for physicians. For the highest cost scenario, we assumed that each HCW had 3 return consultations for the 2-day + reassessment policy and 1.5 return visits for the 7-day policy, that 60% of HCWs received antimicrobial drugs, and that 100% received treatment for symptoms. Staff replacement in the highest cost scenario was assumed to be 100% for nurses and nurse assistants and 80% for physicians.

### Data Sources

The inputs, values, and data sources are presented in Table 1. HC maintains a centralized occupational health database in which sick leave of HCWs since August 2008 is registered by International Statistical Classification of Diseases and Related Health Problems (*13*). The number of HCWs placed on sick leave during August 2008–October 2009 because of diseases of the respiratory system reported as codes J10 (Influenza due to other identified influenza virus) and J11 (Influenza, virus not identified), by using the International Statistical Classification of Diseases and Related Health Problems, 10th Revision, was evaluated (*13*). Cost data were obtained from the hospital administration.

**Table 1:** Evaluation of the effect of ILI-associated absenteeism among HCWs during the pandemic (H1N1) 2009 outbreak, Hospital das Clínicas, São Paulo, Brazil, May–October 2009*

Input	Value	Data source	Assumptions		
			Baseline cost	Lowest cost scenario	Highest cost scenario
No. physicians, nurses, and nurse assistants receiving ILI-associated sick leave	415 for 2-d + reassessment; 169 for 7-d	Centralized occupational health database	NA	NA	NA
Costs					
Medical consultation	R$7.90/ consultation	National Health Care System	1.1 consultations/ HCW for 7-d; 2.5/HCW for 2-d + reassessment	1 consultation/HCW for 7-d; 1.5/HCW for 2-d + reassessment	2.5 consultations/HCW for 7-d; 4/HCW for 2-d + reassessment
Transportation for consultation	R$10.00/round trip	Public transport fare	10% returned for consultation for 7-d 1.5 returns/HCW for 2-d + reassessment	No return visits for 7-d; 0.5 returns/HCW for 2-d + reassessment	1.5 returns for consultations for 7-d; 3 return visits for 2-d + reassessment
Oseltamivir treatment	R$112.40/ treatment	Central pharmacy	Received by 100% of HCWs with suspected influenza	Received by 100% of HCWs with suspected influenza	Received by 100% of HCWs with suspected influenza
Amoxicillin treatment	R$2.40/ treatment	Central pharmacy	Received by 20% of HCWs with suspected influenza.	Received by 5% of HCWs with suspected influenza	Received by 60% of HCWs with suspected influenza
Medication for symptoms	R$2.95/ treatment	Central pharmacy	Received by 70% of HCWs with suspected influenza	Received by 20% of HCWs with suspected influenza	Received by 100% of HCWs with suspected influenza
Diagnostic rt-PCR†	R$100.00/test	Central laboratory	NA	NA	NA
Swab for collecting specimen for rt-PCR	R$0.46/test	Central laboratory	NA	NA	NA
No. HCWs undergoing diagnostic rt-PCR†	244	Central laboratory	NA	NA	NA
No. HCWs hospitalized†	None	Nucleus of Information on Health Care	NA	NA	NA
No. d hospitalization of HCWs†	30 d for 2-d + reassessment; 4 d for 7-d	Direct review of patient records	NA	NA	NA
Daily cost					
Hospitalization	R$1,196.39 for 2-d + reassessment; R$1,871.06 for 7-d	Administration			
Staff replacement	Nurse: R$257.07; nurse assistant: R$167.09; physician: R$858.51	Human resource department	70% of nurses, 70% of nurse assistants, 50% of physicians replaced	30% of nurses, 30% of nurse assistants, 10% of physicians replaced	100% of nurses, 100% of nurse assistants, 80% of physicians replaced
Productivity losses	Nurse: R$307.15; nurse assistant: R$157.18; physician: R$381.23	Human resource department	NA	NA	NA

*ILI, influenza-like illness; HCW, health care worker; 2-d + reassessment, 2-day sick leave plus reassessment every 2 days until asymptomatic policy; 7-d, 7-day sick leave policy; NA, not applicable; R$, Brazilian reals (R$1.80 = US $1.00); rt-PCR, real-time PCR. †For pandemic (H1N1) 2009.

## Results

During June–September 2009, a total of 796 persons with suspected influenza were hospitalized at HC, for which 214 infections were laboratory confirmed as pandemic (H1N1) 2009 (*2*). In July 2009, HCW sick leaves began to increase and peaked in August, when 3% of the workforce received leave for ILI (Table 2). HCWs received 884 ILI-associated sick leaves during August–October 2009, compared with 96 during the same period in 2008 (p<0.00001).

**Table 2:** Total and ILI-associated monthly number, duration, and rates of sick leave by HCWs, Hospital das Clínicas,São Paulo, Brazil, August 2008–October 2009*

Date	Total no. HCWs	ILI-associated			No. sick leaves from all causes	% Sick leaves from ILI
		No. sick leaves	Average duration, d	Days/100 HCWs		
2008						
Aug	17,890	27	1.63	0.25	1,609	1.7
Sep	16,243	33	1.61	0.33	1,931	1.7
Oct	18,064	36	1.36	0.27	1,741	2.1
Nov	18,294	40	1.90	0.42	1,567	2.6
Dec	18,370	47	1.43	0.29	1,555	2.4
2009						
Jan	18,500	31	1.65	0.03	1,586	2.0
Feb	18,697	18	2.11	0.20	1,444	1.2
Mar	18,426	37	1.78	0.36	1,982	1.9
Apr	17,804	30	1.47	0.25	1,815	1.7
May	17,994	40	1.35	0.30	1,884	2.1
Jun	18,102	79	1.66	0.72	1,899	4.2
Jul	18,216	279	3.36	5.14	2,306	12.1
Aug	18,400	548	4.23	12.60	2,716	20.2
Sep	18,544	240	3.37	4.36	2,180	11.0
Oct	18,476	96	2.15	1.11	1,887	5.1

*ILI, influenza-like illness; HCW, health care worker. Includes entire hospital complex.

Of 244 HCWs tested for pandemic (H1N1) 2009, a total of 52 (21%) received positive results. The mean monthly rate of influenza sick leaves per 100 HCWs was significantly higher in the Heart Institute, which had a different sick-leave policy from the other institutes (p<0.0001) (Table 3). The distribution of ILI-associated sick leave of HCWs varied by professional category (Table 4).

**Table 3:** ILI-associated sick leaves for HCWs during pandemic (H1N1) 2009 outbreak, by hospital institute, Hospital das Clínicas, São Paulo, Brazil, May–October, 2009*

Hospital institute†	No. sick leaves	Average duration of sick leave, d	Monthly average no. HCWs in institute	Monthly average no. d of sick leave day/100 HCWs‡	No. hospitalized patients with confirmed pandemic (H1N1) 2009 influenza
Heart	357	5.14	3,507	8.72	34
All other	776	2.89	10,760	3.47	186
Central	436	2.81	5,874	3.48	94
Children’s	65	3.15	1,241	2.75	78
Orthopedics	50	2.18	959	1.89	1
Cancer	145	3.47	1,474	5.69	12
Psychiatry/neurosurgery	42	1.98	679	2.04	1
Radiology	38	3.08	533	3.66	§

*ILI, influenza-like illness; HCW, health care worker. †Excludes the 2 long-term care facilities, the outpatient rehabilitation institute, and the administration building. ‡p<0.0001 comparison between Heart Institute and other Institutes. §The Radiology Institute does not contain inpatient beds.

**Table 4:** ILI-associated sick leaves for HCWs during the pandemic (H1N1) 2009 outbreak, by professional category, Hospital das Clínicas, São Paulo, Brazil, May–October, 2009*

Professional category†	No. ILI-associated sick leaves	Average leave duration, d	Average no. HCWs working in hospital	Total no. sick leave days/total no. HCWs/100 HCWs
Physician	32	5.44	2,284	1.27
Nurse	147	3.88	1,113	8.55
Nurse assistant	416	3.28	3,235	7.03
Nurse technician	72	3.76	520	8.69
Physiotherapist	19	3.79	240	5.00
Nutrition assistant	61	2.52	437	5.87
Laboratory technician	28	4.14	398	4.86
Pharmacy or ECG technician	27	2.19	266	3.70
Janitor, doorman, telephone or elevator operator, etc.	144	2.24	1,339	4.01
Specialized maintenance, e.g., painter, driver, mechanic, plumber, electrician	21	2.95	293	3.53
Administrative officer	97	2.79	1,200	3.76
Administrative assistant	19	4.21	282	4.73

*ILI, influenza-like illness; HCW, health care worker; ECG, electrocardiogram. †Does not include all professional categories.

Three HCWs were hospitalized in HC because of pandemic (H1N1) 2009, resulting in a total of 34 days of hospitalization. One HCW was hospitalized in a private hospital; related costs were covered by private medical insurance and thus not included in our analysis. Total cost in all HC institutes was R$798,051.87 (≈US $443,362). At the Heart Institute (7-day sick leave policy), 169 staff (physicians, nurses, and nurse assistants) received sick leave because of ILI, resulting in a total cost of R$343,082.94 (≈US $190,602). At the remaining 6 institutes (2-day + reassessment policy), a total of 415 staff (physicians, nurses and nurse assistants) received ILI-associated sick leaves, resulting in a total cost of R$454,968.92 (≈US $252,761) (Table 5). Thus, for each HCW on leave, cost was R$1,096.31 (≈US $609.06) for the 2-day + reassessment policy and R$2,030.08 (≈US $1,127.82) for the 7-day policy (Table 6).

**Table 5:** Costs associated with HCW absenteeism during the pandemic (H1N1) 2009 outbreak, by type of sick leave policy, Hospital das Clínicas, São Paulo, Brazil, May–October, 2009*

Cost category	2-d + reassessment, n = 415 HCWs				7-d, n = 169 HCWs		
	Unit cost, R$	No. units	Cost, R$		Unit cost, R$	No. units	Cost, R$
Direct costs							
Diagnosis							
Medical consultation	7.90	1,037.50	8,196.25		7.90	185.90	1,468.61
Real-time PCR	100.00	184.00	18,400.00		100.00	60.00	6,000.00
Respiratory swab	0.46	184.00	84.64		0.46	60.00	27.60
Outpatient care							
Oseltamivir	112.40	415	46,646.00		112.40	169	18,995.60
Antimicrobial drugs	2.40	83	199.20		2.40	34	81.60
Medication for symptoms	2.95	291	858.45		2.95	118	348.10
Hospitalization, d	1,196.39	30.00	35,891.70		1,871.06	4.00	7,484.24
Staff replacement, d							
Physician	858.51	50.50	43,354.76		858.51	17.50	15,023.93
Nurse	257.07	131.60	33,830.41		257.07	166.60	42,827.86
Nurse assistant	167.09	421.40	70,411.73		167.09	419.30	70,060.84
Nonmedical	10.00	1.50	6,225.00		10.00	0.10	169.00
Indirect costs							
Productivity losses, d							
Physician	381.23	101.00	38,504.23		381.23	35.00	13,343.05
Nurse	307.15	188.00	57,744.20		307.15	238.00	73,101.70
Nurse assistant	157.18	602.00	94,622.36		157.18	599.00	94,150.82
Total			454,968.92				343,082.94


*HCW, health care worker; 2-d + reassessment, 2-day sick leave plus reassessment every 2 days until asymptomatic policy; 7-d, 7-day sick leave policy; R$, Brazilian reals (R$1.80 = US $1.00). †Round-trip bus fare for reassessment.

**Table 6:** Costs related to HCW absenteeism resulting from ILI during the pandemic (H1N1) 2009 outbreak, Hospital das Clínicas, São Paulo, Brazil, May–October 2009*

Cost	Baseline cost, R$ (US $)	Lowest cost scenario, R$ (US $)	Highest cost scenario, R$ (US $)
Total	798,051.86 (443,362.14)	617,135.45 (342,853.02)	942,588.49 (523,660.27)
2-d + reassessment	454,968.92 (252,760.51)	352,527.28 (195,848.49)	537,563.93 (298,646.63)
7-d	343,082.94 (190,601.64)	264,608.17 (147,004.54)	405,024.56 (225,013.64)
Per HCW			
2-d + reassessment	1,096.31 (609.06)	849.46 (471.92)	1,295.33 (719.63)
7-d	2,030.08 (1,127.82)	1,565.73 (869.85)	2,396.60 (1,331.44)

*HCW, health care worker; ILI, influenza-like illness; R R$, Brazilian reals (R$1.80 = US $1.00); 2-d + reassessment, 2-day sick leave plus reassessment every 2 days until asymptomatic policy; 7-d, 7-day sick leave policy.

In the lowest cost scenario, total cost was R$617,135.45 (≈US $342,853.02). In the highest cost scenario, total cost was R$942,588.49 (≈US $523,660.27).

During the study period, active surveillance was conducted for hospital-acquired pandemic (H1N1) 2009 infections. No cases of influenza were documented or suspected in patients.

## Discussion

Pandemic (H1N1) 2009 substantially affected HCW absenteeism with significantly higher ILI-associated sick leave during August–October 2009 than during the same period in 2008. The 2-day + reassessment policy was less costly (approximately half the cost per HCW) and as effective for preventing transmission of pandemic (H1N1) 2009 as the 7-day policy because no cases of nosocomial acquisition occurred among patients.

The effect on society of influenza leading to absenteeism is well documented. A systematic review of studies in this field showed a loss of 1.5–4.9 workdays per episode of laboratory-confirmed influenza (*14*), but this review did not focus on HCWs. The impact of influenza epidemics on sickness-associated absence in hospitals is difficult to define. One study involving a hospital in the United Kingdom during 2 influenza seasons (1993–94 and 1996–97) showed that although the number of ILI-associated medical consultations in the population increased markedly during the outbreaks, they did not affect absences from work of hospital staff (*15*). Contrary to that study, our study demonstrated an effect of the pandemic: the number of HCW sick leaves increased greatly during the pandemic (H1N1) outbreak.

The lack of evidence-based policies for HCW sick leaves during influenza pandemics led us initially to consider that recommendations for patients (*9*) applied to HCWs, based on potential transmission for 7 days or even longer if symptoms persist. If we had followed this policy, our workforce would have been substantially reduced; thus our capacity to respond adequately to the pandemic would have been diminished. Because of that, we decided to apply the 2-day + reassessment policy. One of the HC Institutes did not comply with the official policy, which allowed us to compare the policies. Active surveillance for hospital-acquired influenza among patients was essential to validate and allow continued reassessment of the policy. Although costs and cost savings may seem moderate by some standards, it is necessary to remember that the minimum monthly salary in Brazil is R$510, equivalent to ≈US $283.

Because diagnostic testing was not available for HCWs during most of the season, most of the decisions regarding sick leave were based on clinical evaluation, and we could not evaluate whether absenteeism resulted from influenza infections themselves or from other factors, such as the psychological impact of the pandemic and concerns about self and family health related to occupational exposure at work. A few studies have evaluated the effect a pandemic of respiratory disease might have on the attitudes of HCWs (*16**,**17*) and on the distribution of resources within hospitals (*18*). In Australia, only 50% of HCWs questioned said they would come to work during an avian influenza pandemic (*16*), and only 25% said they believed that their department was prepared to handle an influenza pandemic (*17*). In our hospital, diagnostic testing (rt-PCR) was available for HCWs at the end of the season, and only 21% of HCWs tested received positive results. Whether this percentage reflects the previous period, when the test was not available, is not known.

These issues suggest that to mitigate concerns of HCWs, clear infection control strategies and hospital policies to protect HCWs and immediate testing of symptomatic HCWs and patients would reassure the workforce. Thus advanced planning and preparedness for implementation of such policies during epidemics and pandemics is needed.

Our study had several limitations. Part of the costs had to be estimated because of lack of data. We tried to counteract this limitation by estimating highest and lowest cost scenarios. In addition, diagnostic testing was available for HCWs only at the end of the season.

Some positive aspects were observed during the pandemic (H1N1) 2009 outbreak. Awareness increased that HCWs should not work when sick with respiratory infections. Usually HCWs tend to underestimate their health problems and work when ill, placing patients at risk (*7*). Another positive effect was the widespread use of alcohol-gel solutions within and outside the health care environment to halt transmission of infection. The pandemic presented an unprecedented opportunity for education of HCWs, children, and the general public about hand hygiene.

In conclusion, our retrospective study evaluated the effect on HCWs and associated costs of different sick leave policies implemented during the pandemic (H1N1) 2009 outbreak. The 7-day policy was more costly but not any more effective than the 2-day + reassessment policy in preventing transmission to patients. Decisions about HCW sick leave policies during pandemics should account for multiple factors, including effectiveness, cost, and feasibility of implementation during emergency conditions.
